# Molecularly Imprinted Nanomicrospheres as Matrix Solid-Phase Dispersant Combined with Gas Chromatography for Determination of Four Phosphorothioate Pesticides in Carrot and Yacon

**DOI:** 10.1155/2015/385167

**Published:** 2015-04-14

**Authors:** Mengchun Zhou, Nana Hu, Shaohua Shu, Mo Wang

**Affiliations:** College of Plant Science and Technology, Huazhong Agricultural University, Wuhan 430070, China

## Abstract

An efficient, rapid, and selective method for sample pretreatment, namely, molecularly imprinted matrix solid-phase dispersion (MI-MSPD) coupled with gas chromatography (GC), was developed for the rapid isolation of four phosphorothioate organophosphorus pesticides (tolclofos-methyl, phoxim, chlorpyrifos, and parathion-methyl) from carrot and yacon samples. New molecularly imprinted polymer nanomicrospheres were synthesized by using typical structural analogue tolclofos-methyl as a dummy template via surface grafting polymerization on nanosilica. Then, these four pesticides in carrot and yacon were extracted and adsorbed using the imprinted nanomicrospheres and further determined by gas chromatography. Under the optimized conditions, a good linearity of four pesticides was obtained in a range of 0.05–17.0 ng·g^−1^ with *R* varying from 0.9971 to 0.9996, and the detection limit of the method was 0.012~0.026 ng·g^−1^ in carrot and yacon samples. The recovery rates at two spiked levels were in the range of 85.4–105.6% with RSD ≤9.6%. The presented MI-MSPD method combined the advantages of MSPD for allowing the extraction, dispersion, and homogenization in two steps and the advantages of MIPs for high affinity and selectivity towards four phosphorothioate pesticides, which could be applied to the determination of pesticide residues in complicated vegetal samples.

## 1. Introduction

Carrot is among the top ten most economically important vegetable crops in the world, in terms of both area of production and market value [1]; it is generally believed to be rich in vitamins and minerals. Meanwhile yacon (*Smallanthus sonchifolius*) is a perennial herbaceous feverfew, which is native to southern Peru and the Andean plateau of Western Bolivia. In China, yacon has been consumed as health food for many years. Yacon fruit, which is rich in flavonoids, phenolic acids, esters, lipids, and polysaccharides, has stomach-cleansing, detoxification, hypolipidemic, and bactericidal properties [2]. However, carrot and yacon have easily encountered some same plant diseases and pests at southwest area in China, all of which have grown locally; therefore, phosphorothioate agrochemicals have been often used in the two root crops. Among the pesticides, organophosphate species are the most used due to their high insecticidal activity and relatively low persistence. Furthermore, the application of organophosphate pesticides (OPPs) during crop cycles could contaminate surface and ground soil and can be toxic to aquatic microorganisms, humans, root crops, and other plants [3]. Therefore, a rapid, convenient, accurate, and sensitive method is required to be developed to monitor the concentration of organophosphorous pesticides in carrot and yacon samples. It is especially more important to monitor phosphorothioate pesticides in time since they are the major organophosphate pesticides in carrot and yacon samples. In the past decades, a large number of screening methods have been used for the determination of organophosphorus pesticides, such as thin layer chromatography [4], gas chromatography [5], biosensor [6], and chromatography-mass spectrometry [7]. Among them, gas chromatography (GC) is one of the most classical and universal techniques at present due to the very low limits of detection, but GC determination is insufficient for the analysis of trace pesticide residues [8]. Consequently, an effective sample preparation procedure before GC analysis is very significant and valuable.

Sample extraction, purification, and enrichment of pretreatment procedure are very crucial steps in an analytical process when the target compounds are detected. Currently, there have been a great number of the traditional pretreatment methods in pesticide residue analysis, such as Soxhlet extraction, liquid-liquid extraction (LLE), solid-phase extraction (SPE), and ultrasonic extraction. These methods not only require lots of organic solvents, but also need plenty of time; moreover, they might cause environmental pollutions. Compared with the traditional pretreatment methods, a promising technique named matrix solid-phase dispersion (MSPD) has been developed and extensively applied in recent years, which enables extraction and cleanup to be performed in one single step [9]. Application of MSPD in sample analysis can greatly reduce the analysis time and solvent because no elution of the SPE column is needed, which can evidently decrease the cost of analysis [10]. However, because of the lack of selectivity and ideal purification efficiency, the common dispersants in matrix solid-phase dispersion (C18, N-ethylenediamine propyl silane, silica, florisil, etc.) are easily subjected to the interference of nontarget compounds with similar characteristics [11]. Therefore, further improving the adsorption and selectivity of matrix solid-phase dispersion is still very crucial and significant. The molecularly imprinted polymers (MIPs) are very ideal matrix solid-phase dispersant, which can be successfully applied in pretreatment process of samples.

Molecularly imprinted polymers (MIPs) are artificially synthesized macromolecular materials, which are highly cross-linked polymers and are able to recognize the target molecules by imprinting the molecules during polymer synthesis through covalent or noncovalent interactions [12]. So far, MIP has been researched and utilized extensively in such fields as biochemical separation [13], chiral resolution [14, 15], enzyme catalysis [16, 17], chromatographic analysis [18], biosensors [19], drug delivery [20], and so on. In recent years, MIPs have been extensively used for the selective enrichment and pretreatment of target compounds which exist in a complex matrix sample. It is a new trend that MIPs are used as selective MSPD sorbents to achieve simultaneous extraction and purification of analytes, which can significantly reduce the labor and cost of the analysis. In addition, MIPs technology has also been gradually applied to the field of environmental analysis with the rapid development of analytical methods [21]. However, the conventional techniques used to prepare MIPs most often result in a poor site accessibility of the MIPs to the target molecules. MIPs especially prepared by the method of traditional bulk polymerization can only selectively recognize the template molecules, but their adsorption capacities toward other analytes are low and weak. Furthermore, a template leakage of MIP is always observed in its actual applications [22, 23]. Therefore, their applications in the multiresidue analysis of pesticides are limited to a great extent. Recently, a new molecular imprinting technique called “grafting from special materials” technique has been developed to overcome this drawback [24]. In this technique, the initiating MIP groups are immobilized on the surface of a solid support, and the grafted MIPs were propagated from the surface of the solid supports during the course of polymerization. Meanwhile, MIP thin layers with high graft density and nanometer thickness can be prepared on the surface of a solid support by using the “grafting from special materials” technique. Therefore, novel nanosilica microspheres were used as solid supporting materials in this study.

The aim of this study is to employ tolclofos-methyl molecularly imprinted nanomicrospheres as a dispersant of MSPD based on supporting materials of nanosilica microspheres, which was used for the rapid screening and adsorbing of four phosphorothioate pesticides from the extracted solution of carrot and yacon. The nanomicrospheric imprinted polymers of tolclofos-methyl were synthesized via a method of grafting technique on the surface of nanosilica. The obtained MIPs were used as the adsorbent of matrix solid-phase dispersion to extract and preconcentrate the four phosphorothioate pesticides from the extracted solution of the carrot and yacon samples. It especially was very significant and novel in process of separation of the sample that magnesium chloride was used for improving adsorbance of MIP nanomicrospheres towards pesticides in a process of pretreatment of the carrot and yacon samples. Then the factors affecting the preconcentration and separation of the analytes were discussed in detail and the applicability of this method was evaluated.

## 2. Materials and Methods

### 2.1. Materials and Reagents

The pure product of tolclofos-methyl with purity of 99.7 ± 0.5% and the single standard solution of four phosphorothioate pesticides (1000 *μ*g·mL^−1^) (phoxim, tolclofos-methyl, chlorpyrifos, and parathion-methyl) were purchased from Aladin Reagent (Beijing, China). Ethyl acetate, acetonitrile, methylene chloride, methanol, and ethyl alcohol were also purchased from Aladin Reagent which all were of chromatographic grade. N-Ethylenediamine propyl silane, nanosize silica (50–100 nm), magnesium sulfate, magnesium chloride, acetic acid, and trifluoroacetic acid were purchased from Sigma Company which all were of analytical grade. Ethylene glycol dimethacrylate (EGDMA, 98% purity), *α*-methacrylic acid (MAA, 99% purity), gamma-(methacryloyloxy) propyl trimethoxy silane, and 2,2-azobisisobutyronitrile were also purchased from Sigma company, all of which were of analytical grade. 10% magnesium chloride (MgCl_2_) was prepared with pure water. For the standard curve, the standard solution was diluted to 5.0, 2.5, 1.0, 0.25, and 0.05 *μ*g·mL^−1^.

### 2.2. Instruments and Analytical Conditions

Analysis of four phosphorothioate pesticides was performed using an Agilent 7890 gas chromatograph (Agilent, CA, USA) equipped with a nitrogen phosphorus detector (NPD). The separation was performed on an Agilent DB-35ms column (30 m × 320 *μ*m, 0.25 *μ*m). Nitrogen was used as the carrier gas at a constant flow rate of 1.0 mL/min. Meanwhile, hydrogen was used as the burning gas at a constant flow rate of 3 mL/min and the auxiliary burning gas was air at 60 mL/min. The injection volume was 1.0 *μ*L, and the injection port temperature was held at 200°C at the splitless mode. The detector temperature was held at 300°C. The oven temperature was programmed as follows: 150°C held for 1.0 min, and then the temperature was increased to 280°C at a rate of 10°C/min and held for 5 min.

The morphological features of the imprinted polymer grain were examined under a JEOL JSM-5610LV scanning electron microscope (JEOL, Tokyo, Japan). Infrared analyses between 400 and 4000 cm^−1^ of the MIP- and NIP-imprinted polymer grains were performed in a VERTEX 70-IR spectrometer (Bruke, Germany) where KBr was used to prepare the samples.

### 2.3. Preparation of Molecularly Imprinted Polymers

#### 2.3.1. Alkylating Synthesis of Gamma-(Methacryloyloxy) Propyl Trimethoxy Silane Functionalized Nanosilica

Nanosilica was pretreated in order to eliminate any surface contaminants and to activate the surface silanol groups for silanization. In a typical experiment, 15 g silica gel was pretreated by soaking in 10% HCl solution for 8 h and was rinsed thrice by deionized water.

Active nanosilica was prepared as follows: 10 g of pretreated nanosilica, 300 mL of acetic acid, and 35 mL of ammonia water were added to 140 mL of pure water; then the mixture was carried out under nitrogen atmosphere at 45°C for 1 h with magnetic stirring. After centrifugation at 8000 rpm for 10 min at 4°C, the sediment of active nanosilica was obtained and washed thrice with absolute ethyl alcohol, rinsed by deionized water, and then dried in vacuum at 60°C before use.

Dried active nanosilica (5 g), gamma-(methacryloyloxy) propyl trimethoxy silane (20 mmol), and 10 mL absolutely dry toluene were introduced into a conical flask under the atmosphere of nitrogen. At a constant temperature of 90°C and with continuous stirring, the reaction was allowed to proceed for 20 h. The particles were then separated from the mixture via centrifugation. The product was washed with toluene for five times and then washed with methanol for five times in order to remove the excessive gamma-(methacryloyloxy) propyl trimethoxy silane. At the end, the obtained gamma-(methacryloyloxy) propyl trimethoxy silane nanosilica was dried under vacuum at room temperature.

#### 2.3.2. Preparation of Prepolymerization Mixture and Initiator Solution

1.0 mmol of tolclofos-methyl was dissolved into 40 mL acetonitrile, followed by the addition of 15 mmol of ethylene glycol dimethacrylate (EGDMA) and 3 mmol g of *α*-methacrylic acid (MAA). The mixture was sonicated for 10 min and then degassed for 20 min using nitrogen, and the sealed system was heated in a water bath shaker at 40°C for 22 h under nitrogen protection to produce prepolymerization mixture. Preparation of initiator solution was carried out by adding 0.09 g of 2,2-azobisisobutyronitrile into 1.5 mL of acetonitrile.

#### 2.3.3. Preparation of Imprinted Polymer Nanomicrospheres

Molecularly imprinted polymer nanomicrospheres were prepared by synthesis of tolclofos-methyl imprinted polymers on the surface of gamma-(methacryloyloxy) propyl trimethoxy silane nanosilica. 5.0 g of activated gamma-(methacryloyloxy) propyl trimethoxy silane nanosilica was mixed fully with prepolymerization mixture and then 0.5 mL initiator solution was added into this prepolymerization mixture. After the system was degassed for 20 min using nitrogen, the sealed system was heated in a water bath shaker at 60°C for 18 h under nitrogen protection to produce polymerized mixture. Then the resulting nanomicrospheres were cooled to room temperature.

The obtained polymer nanomicrospheres were washed with methanol-acetic acid (v/v, 9 : 1), methanol, and acetonitrile solution till no template could be detected. And then the solid was dried in vacuum at 40°C, resulting in a complex of tolclofos-methyl imprinted polymers with nanosilica. Synthesis of nonimprinted polymer microspheres on the surface of nanosilica was carried out in a similar manner but without the addition of template. Finally, a small part of the particles of sediment was scanned by JEOL JSM-5610LV electron microscope.

### 2.4. Effect of the Amount of Cross-Linking Agent on the Adsorbing Performance of MIPs

This test was performed according to Section 2.3. Meanwhile, the added amounts of tolclofos-methyl, MAA, and activated nanosilica gel were, respectively, 1.0 mmol, 3.0 mmol, and 5 g among them. But the added amount of crosslinking agent EGDMA on preparation of MIPs was set to different adding quantity (3, 6, 9, 12, 15, 18, and 21 mmol). After the seven species of different MIPs were synthesized, 50 mg microsphere particles of MIPs were, respectively, weighed in 5 mL test tube, and 1 mL methanol was added to each tube. Then, 400 *μ*L single standard solutions (25.0 *μ*g·mL^−1^) of tolclofos-methyl and 0.2 mL 10% magnesium chloride (MgCl_2_) were, respectively, added to each test tube. And then this group's solutions were, respectively, metered volume to 10 mL using methanol and vibrated for 3 h. The liquid was centrifuged at 10000 r/min under the circumstance of 4°C.

Then clear solution was discarded, and the sediment was taken out. 10 mL acetonitrile- trifluoroacetic acid (99 : 1, v/v) was added to extract sediments by vibrating for 30 min and then the acetonitrile phase was separately filtered and the filtered acetonitrile solution was evaporated to dryness and dissolved in 1.0 mL dichloromethane of chromatographic grade again. The dichloromethane solution was filtered through a 0.22 *μ*m membrane and analyzed by GC. Finally, an adsorption curve of the different MIPs was drawn.

### 2.5. Adsorption Kinetic Experiments

50.0 mg of the MIPs and NIPs was placed in vial containing 5 mL of 5.0 *μ*g·mL^−1^ of four phosphorothioate pesticides in methanol and shaken for 1–6 h, respectively. Then the MIPs or NIPs were rapidly removed by a filter with 0.45 *μ*m pore size, and the remaining pesticides in obtained solutions were analyzed by GC to get the information about the adsorption kinetics of MIPs and NIPs. The adsorption amount (*Q*) of analyte was calculated according to *Q* = (*W*_0_ −* W*_1_)/*W*, where* W*_0_ and* W*_1_ (*μ*g) are the initial amount and the residual amount of the analyte at different time, respectively, and* W* (g) is the amount of the polymers.

### 2.6. Absorbability of MIP and NIP

50 mg microsphere particles of MIP were, respectively, weighed in two-group 5 mL test tube (group A and group B), and 1 mL methanol was added to each tube. Then, 400 *μ*L mixed standard solutions (1.0, 5.0, 10.0, 25.0, 50.0, and 100.0 *μ*g·mL^−1^) of four phosphorothioate pesticides and 0.2 mL 10% magnesium chloride (MgCl_2_) were, respectively, added to each test tube of group A. By contrast, only 400 *μ*L mixed standard solutions (1.0, 5.0, 10.0, 25.0, 50.0, and 100.0 *μ*g·mL^−1^) of four phosphorothioate pesticides were, respectively, added to each test tube of group B. Meanwhile, the procedure same as group A was performed for the NIPs, which was designated as group C. And then this three-group solution was, respectively, metered volume to 10 mL using methanol and vibrated for 3 h. The liquid was centrifuged at 10000 r/min under the circumstance of 4°C.

Then clear solution was discarded, and the sediment was taken out. 10 mL acetonitrile- trifluoroacetic acid (99 : 1, v/v) was added to extract the three groups of sediments by vibrating for 30 min; then the acetonitrile phase was separately filtered and the filtered acetonitrile solution was evaporated to dryness and dissolved in 1.0 mL dichloromethane of chromatographic grade again. The dichloromethane solution was filtered through a 0.22-*μ*m membrane and analyzed by GC, and an adsorption curve of the group was drawn.

### 2.7. Selectivity Experiment

The recognition studies of adsorption capacity were performed with iprobenfos which is the structurally similar compound of the four pesticides.

The mixed standard solution of the four pesticides (phoxim, tolclofos-methyl, chlorpyrifos, and parathion-methyl) and iprobenfos, each of 5 mg·L^−1^, were prepared in 10 mL of the methanol. And then 20 mg MIPs was added to this liquid phase. The mixture was shaken for 3 hours at room temperature. After the solutions had been centrifuged, the concentrations of the four pesticides and iprobenfos in the supernatants were determined by GC.

The same procedure was performed for the NIPs.

### 2.8. Sample Preparation

In this experiment, after fresh carrot and yacon samples were homogenated through tissue homogenizer, which were collected from supermarket of Huazhong Agricultural University in Wuhan city. An aliquot of 0.2 g of samples was placed in an agate mortar and grounded firmly with the pestle. Then, 0.3 g of MIPs and 0.05 mL 10% magnesium chloride (MgCl_2_) prepared with pure water were added to the agate mortar. Intimate contact between the sorbent and the sample was obtained by pounding with the pestle for some minutes to produce a homogenous packing material for MSPD. The homogenized mixture was then added into a 6 mL solid-phase extraction (SPE) column, which contained a polyethylene frit at the bottom and compacted by another frit on the top. After being packed for 5 min, 0.3 mL of methanol-water (1 : 2, v/v) and 0.1 mL of 10% magnesium chloride (MgCl_2_) were added into column; the column was shelved and incubated for a period of 3 h at room temperature. Then, the column was rinsed with 5.0 mL of methanol-water (1 : 9, v/v) and eluted with 6.0 mL of acetonitrile-trifluoroacetic acid (99 : 1, v/v). The eluent was then evaporated to dryness under nitrogen and dissolved in 1.0 mL dichloromethane of for further GC analysis.

### 2.9. Spiked Sample Preparation

To test the accuracy of the MIP-GC method, the samples of blank carrot and yacon were, respectively, spiked with standard substance of four phosphorothioate pesticides, which all were collected from farmland of Huazhong Agricultural University. Prior to spiking, the blank carrot and yacon were ensured to be free of the four phosphorothioate pesticides. Briefly, 0.01 mL of mixed standard solution (0.01 and 0.05 mg·L^−1^) containing 0.1 and 0.5 ng of phosphorothioate pesticides in acetonitrile were added into 0.2 g of homogenated blank carrot and yacon sample, respectively. After being incubated for a period of 1.0 h, the spiked samples were extracted and cleaned according to the above sample preparation procedure. The treatment for each sample was repeated for five times. Then the resulting extractions were detected by GC.

In addition, as a comparison with MIPs, 100 mg commercial N-ethylenediamine propyl silane was also used as dispersant of MSPD to clean and enrich the above four pesticides in blank carrot and yacon samples by above same spiked sample procedure. Furthermore, the treatment for each sample was also repeated for five times.

## 3. Results and Discussion

### 3.1. Preparation of MIPs and NIPs

An adequate molar ratio of template to functional monomer is essential to successful imprinting [25]. In most cases, the molar ratio of template to functional monomer could be approximately set from 1 : 3 to 1 : 5 (the molar ratio was at 1 : 3 in this experiment). *α*-Methacrylic acid was selected as the functional monomer because it is favorable for hydrogen bond or ionic bond interaction in the porogen prior to polymerization. A stable complex between the template and functional monomer was formed in the imprinting process.

### 3.2. Effect Evaluation of the Amount of Cross-Linking Agent on the Adsorbing Performance of MIPs

**Figure 1:**
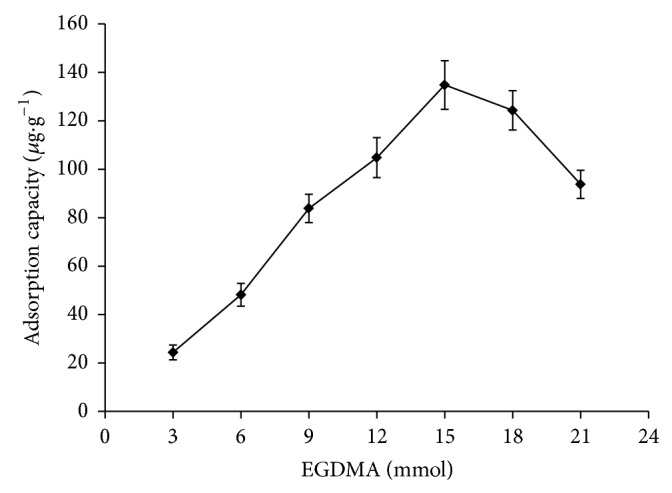
Effect of the amount of cross-linker on the adsorption capacity of MIPs.

The effect of added amount of crosslinking agent EGDMA on adsorption amount of MIPs towards template molecule tolclofos-methyl was investigated with different adding quantity. And the added amounts of tolclofos-methyl, MAA, and activated nanosilica gel were, respectively, 1.0 mmol, 3.0 mmol, and 5 g among them. As is shown in Figure 1, the adsorption capacity reached maximum value at 15 mmol of the amount of crosslinking agent (EGDMA); furthermore, the adsorption capacity increased at the start with the growth in the amount of crosslinking agent and decreased after maximum value. The phenomenon might be attributed to following reason; MIP formed at start had been only poor mechanical strength and chemical stability due to the dosage of cross-linking agent [26]. At the same time, too little dosage of cross-linking agent might cause deficiency of quantity of the three-dimensional cavity structure, but too much dosage of cross-linking agent might lead to too tight MIPs network structure. It might make its accessibility of recognized site to get weak; therefore, it might make MIP nanomicrospheres not easily to spread and to result in the decrease of the adsorption capacity of imprinting molecule.

### 3.3. Polymer Synthesis and Characterization

**Figure 2:**
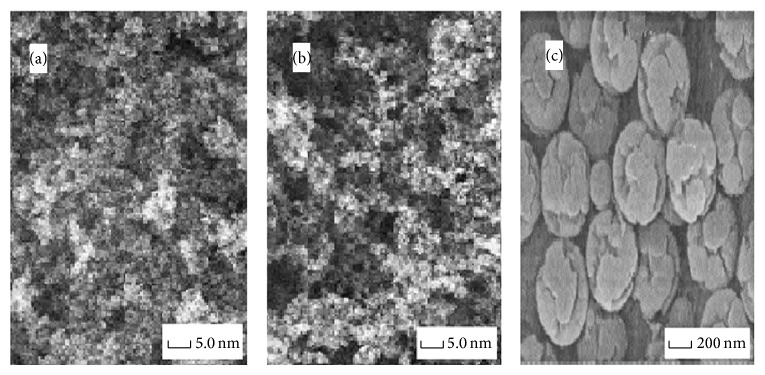
Micrographs of the molecularly nonimprinted microspheres (a) and molecularly imprinted nanomicrospheres (b, c).

Under the optimized synthesis conditions, MIPs were produced in a mold, such as a glass bottle or tube. The polymers were subsequently filtered and cleared either manually or mechanically. This method is simple and fast, yielding polymers that can be used as extracted sorbents in matrix solid-phase dispersion.  Figure 2 shows the environment and morphology of the synthesized polymers, which have a lacunose structure. The morphology and size were characterized by scanning electronic microscopy (SEM) with a JEOL JSM-5610LV scanning electronic microscope. The picture shows that the number of cavities of NIP nanomicrospheres (Figure 2(a)) seems to be far less than that of MIP nanomicrospheres (Figure 2(b)). Furthermore, the surface of NIP microspheres also seems to be smoother than that of MIP nanomicrospheres.  Figure 2(c) indicates that the resulting imprinted polymer nanomicrospheres were monodisperse and showed a uniform spherical morphology with a diameter of about 200 nm.

**Figure 3:**
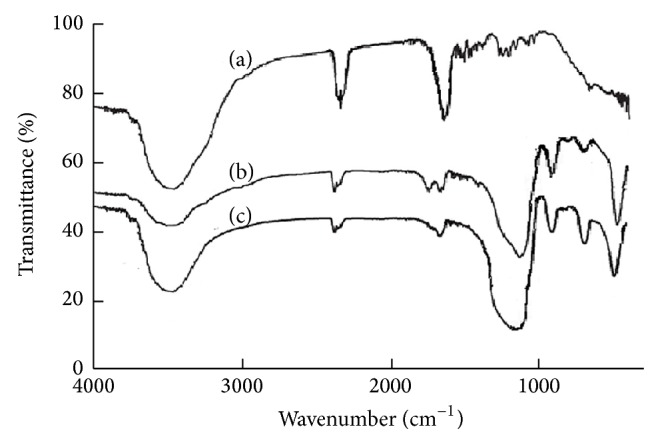
FT-IR spectra of molecularly imprinted nanomicrospheres (a), molecularly nonimprinted microspheres (b), and gamma-(methacryloyloxy) propyl trimethoxy silane-silicon dioxide (SiO_2_) (c).

Figure 3 shows the infrared spectra of MIP and NIP molecularly imprinted polymers. IR spectra (Figure 3(a)) represent MIP nanomicrospheres. IR spectra (Figure 3(b)) and spectra (Figure 3(c)), respectively, represent NIP microspheres and gamma-(methacryloyloxy) propyl trimethoxy silane-silicon dioxide (SiO_2_) microspheres. The MIP and NIP molecularly imprinted polymers were washed with methanol/acetic acid (90 : 10, v/v), methanol, and acetonitrile solution. As shown in Figure 3, for the FT-IR spectra of the imprinted and nonimprinted polymers in Figure 3(a) (MIP) and Figure 3(b) (NIP), the features around 3595 cm^−1^ indicate a −OH vibration. This shift can be attributed to the reaction of the −OH group of *α*-methacrylic acid. For the imprinted polymers in Figure 3(a), the observed features around 2436 cm^−1^ indicate a C=O stretch. Furthermore, for the FT-IR spectra of imprinted polymers in Figure 3(a) (MIP), the strong features around 1713 cm^−1^ indicate the presence of a P=S bond, and the shift in the position of this stretch can be attributed to the hydrophobic interaction between the P=S group of tolclofos-methyl and the –OH group of *α*-methacrylic acid [27]. These results demonstrate that tolclofos-methyl had been reacted with *α*-methacrylic acid and that nanomicrosphere polymers had been synthesized. The FT-IR spectra of the imprinted and nonimprinted polymers after extraction are similar, indicating that the template molecule was completely removed from the imprinted polymers. In Figures 3(b) and 3(c), the features around 1100 cm^−1^ and 476 cm^−1^ were evidently observed in the FT-IR spectra of the nonimprinted polymers and gamma-(methacryloyloxy) propyl trimethoxy silane-silicon dioxide (SiO_2_) microspheres, indicating a Si–O vibration, while these features were almost unobservable in imprinted polymers (Figure 3(a)), suggesting that tolclofos-methyl had been successfully imprinted in polymeric nanomicrospheres. Furthermore, the two characteristic peaks in 1713 cm^−1^ and 2436 cm^−1^ of Figure 3(a) are all evidently stronger than those of Figures 3(b) and 3(c), suggesting that tolclofos-methyl was successfully imprinted into the surface of nanosilica. Therefore, the two characteristic peaks of nanosilica were observably weakened and decreased in Figures 3(b) and 3(c).

### 3.4. Evaluation of Adsorption Kinetic Experiments

**Figure 4:**
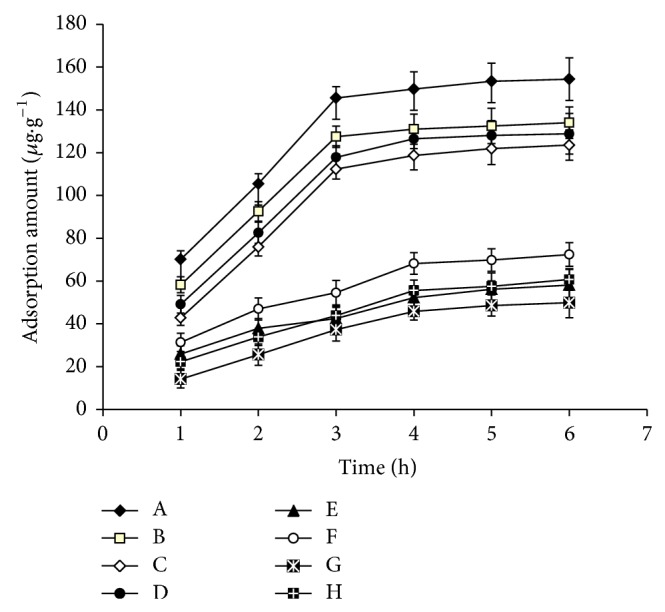
Adsorption kinetics of MIPs and NIPs to phosphorothioate pesticides (A, B, C, D, E, F, G, and H—adsorption kinetics of tolclofos-methyl (A), parathion-methyl (B), chlorpyrifos (C), phoxim (D) on MIPs (A, B, C, and D) and tolclofos-methyl (E), parathion-methyl (F), chlorpyrifos (G), and phoxim (H) on NIPs (E, F, G, and H)).

To investigate the adsorption kinetics of MIPs and NIPs, equilibrium time was investigated in this work [28]. It can be seen from Figure 4 that the amount of four pesticides adsorbed onto MIPs increased with longer hours and nearly reached saturation state within 3.0 h, as well as the NIPs. The high adsorption rate within 3.0 h might result from the prior and efficient adsorption of recognition sites at the surface of MIPs, and the adsorption equilibrium of the polymers towards pesticides could be achieved in 3.0 h. Thus, the adsorption time was set for 3.0 h in the following adsorption capacity and selectivity experiments.

### 3.5. Evaluation of Adsorption Capacity and Selectivity of the Imprinted Polymer Nanomicrospheres

**Figure 5:**
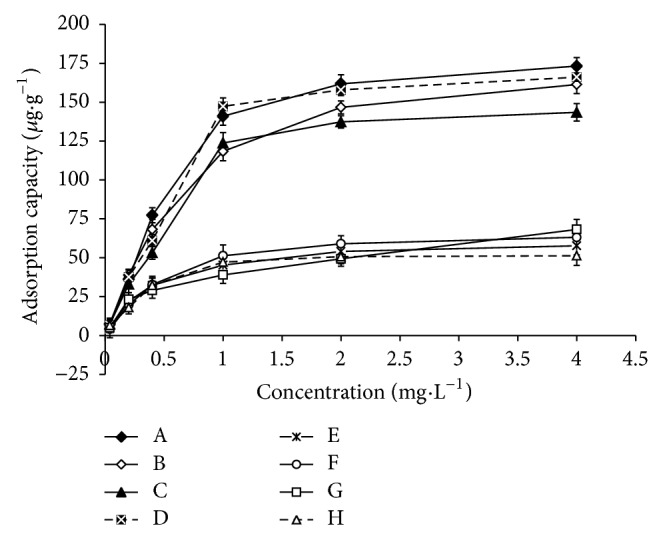
Adsorption isotherms curves of four pesticides on molecularly imprinted nanomicrospheres (MIPs) and molecularly nonimprinted microspheres (NIPs) (A, B, C, D, E, F, G, and H—adsorption curves of tolclofos-methyl (A), phoxim (B), chlorpyrifos (C), parathion-methyl (D) on MIPs (A, B, C, and D) and phoxim (G), parathion-methyl (F), chlorpyrifos (E), and tolclofos-methyl (H) on NIPs (E, F, G, and H)).

The isothermal adsorptions of the imprinted and no-imprinted polymer nanomicrospheres are plotted in Figure 5. The data show that the adsorption capacity of molecularly imprinted or nonimprinted polymer nanomicrospheres increased together with the raise of the initial concentrations of the four phosphorothioate pesticides. However, the imprinted polymer nanomicrospheres exhibited a stronger memory function and a higher adsorption capacity for the four phosphorothioate pesticides than the nonimprinted polymers. The adsorption capacity of the imprinted polymer (173.2 *μ*g·g^−1^) was about 3.4-fold that of the nonimprinted polymer (51.3 *μ*g·g^−1^) at a 4 mg·L^−1^ concentration of template tolclofos-methyl (Figures 5(A) and 5(H); Table 1). The adsorption capacity of the imprinted polymer (143.5 *μ*g·g^−1^) was about 2.5-fold that of the nonimprinted polymer (57.7 *μ*g·g^−1^) at a 4 mg·L^−1^ concentration of chlorpyrifos (Figures 5(C) and 5(E); Table 1). The static adsorption capacity of molecularly imprinted nanomicrospheres toward the four phosphorothioate pesticides was approximately twice to triple that of the nonimprinted polymers. Furthermore, the adsorption capacity of the molecularly imprinted nanomicrospheres towards tolclofos-methyl was maximal in the four phosphorothioate pesticides because tolclofos-methyl was used as the template. In addition, the adsorption capacity of the molecularly imprinted nanomicrospheres towards chlorpyrifos was the minimum in the four phosphorothioate pesticides owing to the maximal relative molecular mass of chlorpyrifos compared with other three pesticides. The results reveal that molecularly imprinted nanomicrospheres of tolclofos-methyl have a higher adsorption capacity and selectivity for the four kinds of phosphorothioate pesticides than nonimprinted polymers. Furthermore, as shown in Figure 5, the diversity of adsorbance of the MIPs for the four pesticides (A, B, C, and D) was more evident than that of NIPs for the four pesticides (E, F, G, and H), implying that the selectivity of MIPs is evidently better than that of NIPs for the four pesticides.

In addition, magnesium chloride was used in determination of the adsorbance of MIPs and NIPs for the four pesticides at group A and group C. By contrast, magnesium chloride was not added at group B. Then, the adsorbance of three groups was shown in Table 1. It was very evident contrast that adsorbance of A group seems to be consistently one-third higher than that of group B because of the effect of magnesium chloride. The results suggest that magnesium chloride can improve the adsorbance of imprinted polymer nanomicrospheres remarkably in pretreatment program of samples. That magnesium chloride can facilitate the extraction (adsorption) of the analytes may be due to that magnesium chloride can decrease the solubility of phosphorothioate pesticides in the solvent and increase the activity of MIPs.

### 3.6. Selectivity of the Imprinted Sorbent

The structurally similar compound iprobenfos was used as a typical species that compared with representational four pesticides (phoxim, tolclofos-methyl, chlorpyrifos, and parathion-methyl) in a recognition study. The distribution coefficient (*K*_*d*_), the selectivity coefficient of the sorbent (*k*), and the relative selectivity coefficient (*k*′) were obtained in these comparative experiments (Table 2). *K*_*d*_ indicates the affinity of the sorbent for a particular substance; *k* indicates how selective the sorbent is for one of the substances when it is exposed to several substances; *k*′ indicates how selective a sorbent is for a particular substance when compared with the selectivity of a different sorbent and *Q* means the adsorbance value of specific binding sites of the imprinted polymers. These factors were calculated using [29]

(1) $$\begin{array}{c} K_{d}=\left(C_{i}-C_{f}\right)\times \frac{V_{s}}{\left(C_{f}\times M_{p}\right)},\\ K=\frac{K_{d}\;\left(\text{each}\;\text{one}\;\text{of}\;\text{four}\;\text{pesticides}\right)}{K_{d}\;\left(\text{iprobenfos}\right)},\\ K^{\prime }=\frac{K_{\text{imprinted}}}{K_{\text{nonimprinted}}},\\ Q=\Delta C\times \frac{V_{s}}{M_{p}}\;\left(\Delta C=C_{f\text{-nonimprinted}}-C_{f\text{-imprinted}}\right), \end{array}$$

where *C*_*i*_ and *C*_*f*_ represent the initial and final concentrations of the each pesticide adsorbed; *V*_*s*_ represents volume of solution. *M*_*p*_ represents mass of polymer; Δ*C* represent the different value of each pesticide's final concentrations of the MIPs and NIPs.

The tolclofos-methyl imprinted polymer adsorbed twice to fourfold as much four pesticides as iprobenfos. The *k* (every one of four pesticides/iprobenfos) values of the imprinted polymer sorbent were between 0.8 and 2.2 in 5 mg·L^−1^, which were evidently larger than that of the NIP sorbent (0.5~1.1). The results show that the imprinted polymer sorbents of tolclofos-methyl have a higher selectivity and *Q* value for four pesticides than for iprobenfos, which is structurally very similar to the four pesticides. The *k*′ value was greater than 1, which showed that the MIP sorbent had a higher selectivity than the NIP sorbent.

As expected, iprobenfos was retained to some extent in the active centers of MIPs, but not in a quantitative way and the repeatability was poor. There were specific and nonspecific binding sites in the MIPs, but in the NIPs there were only nonspecific binding sites. The MIPs show a higher affinity for four pesticides owing to the specific sites. It is also obvious that the specific recognition sites are mainly complementary to the template in terms of size and shape. Furthermore, the *K*_*d*_, *K*, *K*′, and *Q* values of tolclofos-methyl were highest among the four pesticides due to being used as template molecule; at the same time, the *K*_*d*_, *K*, *K*′, and *Q* values of chlorpyrifos were lowest among the four pesticides owing to being maximal relative molecular mass.

### 3.7. Validation of the Method

#### 3.7.1. Evaluation of Method Performance

**Figure 6:**
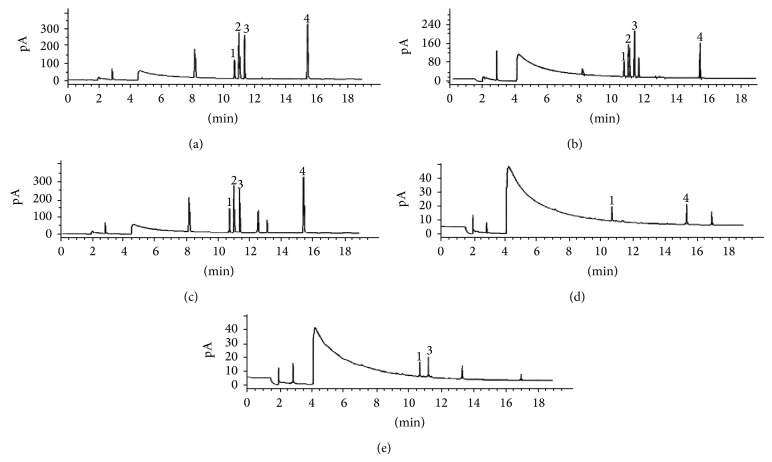
Chromatograms of the standard mixture solution of four pesticides in 25 ng·mL^−1^ (a), the spiked sample in 2.5 ng·g^−1^ of four pesticides after preconcentration by N-ethylenediamine propyl silane sorbent (b), the spiked sample in 2.5 ng·g^−1^ of four pesticides after preconcentration by MIPs sorbent (c), the actual carrot sample (d), and the actual yacon sample (e). Peak identification: 1, tolclofos-methyl; 2, parathion-methyl; 3, chlorpyrifos; 4, phoxim.

The analytical figures presented in this method for the simultaneous determination of four phosphorothioate pesticides were estimated under optimal conditions. This study defined the lowest concentration used in the calibration curve, 10 ng·g^−1^, to be the limit of detection (LOD) for each pesticide. Detection limits were verified by injection of the samples prepared at 10 ng·g^−1^ to ensure that discernible peaks had a signal-to-noise ratio ≥3. The results indicated that the limit of detection (LOD) (*S*/*N* = 3) of this method for the four phosphorothioate pesticides was in the range of 0.012~0.026 ng·g^−1^ (Table 3). As shown in Figure 6(a), the four target compounds were entirely separated by Agilent DB-35ms column (30 m × 320 *μ*m, 0.25 *μ*m). Furthermore, the linear ranges of the calibration graph were all between 0.05 and 17.0 ng·g^−1^ (*R* > 0.9971).

At present, only several relevant literatures have been reported [30, 31]. For instance, some scholars such as Wang et al. made use of homemade fiber and gas chromatography to develop a method for the determination of five organophosphorous pesticides in pakchoi samples in 2008 [30], which indicated that the LOD of methyl parathion was 0.05 ng·g^−1^. This LOD value is observably higher than that (0.012 ng·g^−1^) of parathion-methyl in this paper. And linearity range of this literature was also more narrow than that of this paper, which showed that the linearity range of parathion-methyl was 0.6~60 ng·g^−1^. This range is observably higher and narrower than that (0.05~10 ng·g^−1^) of parathion-methyl in this paper.

For another example, some other scholars such as Wong et al. employed capillary gas chromatography-mass spectrometry and flame photometric detection to build a method for the determination of organophosphorus pesticides in ginseng root in 2007 [31]. By contrast, the LOD of pesticides in their study was 5~500 ng·g^−1^, which is also obviously higher than the LOD of pesticides in this experiment. Therefore, the method of using GC technique and condition can evidently cut down the LOD value of the determinand, which obviously improves the sensitivity of the method and decreases the cost of determination. And linearity range of the method was also obviously broadened owing to novel sample pretreatment and GC technique.

#### 3.7.2. Applicability Assessment of the Method

To evaluate the applicability of the this method, the selectivity and enrichment of MIP matrix solid-phase dispersion and N-ethylenediamine propyl silane matrix solid-phase dispersion were compared by the spiking of four phosphorothioate pesticides at the levels of 0.5 and 2.5 ng·g^−1^ in the food samples; and then the spiked samples were extracted and analyzed (Figures 6(b) and 6(c)). The results show that the traditional method of N-ethylenediamine propyl silane matrix solid-phase dispersion has good concentration effect but poor selectivity for the four phosphorothioate pesticides (Figure 6(b)). Therefore, the enrichments of phoxim, tolclofos-methyl, chlorpyrifos, and parathion-methyl were almost the same when the traditional adsorbent was used (Figure 6(b)). By contrast, the imprinted matrix solid-phase dispersion (Figure 6(c)) shows a better concentration effect and selectivity for the four phosphorothioate pesticides than the traditional N-ethylenediamine propyl silane matrix solid-phase dispersion. These results clearly indicate that imprinted polymers are more suitable to be used as matrix solid-phase dispersants for the scientific separation and extraction of pesticides.

For each concentration, five replicate measurements were performed, and good recovery rates between 85.4 and 105.6% were obtained by MIPs when MIPs were used for extracting pesticides in carrot and yacon (Tables 4~5). The recovery precision (RSD) for five replicate extractions of the spiked samples was less than 9.6%. By contrast, low recovery rates between 63.0 and 87.6% were obtained when N-ethylenediamine propyl silane was used for extracting pesticides in carrot. The obtained results suggest that MIP is better than N-ethylenediamine propyl silane for extracting pesticides in carrot and yacon sample. Furthermore, there was an interference peak to be glued to target peak 2 (Figure 6(b)), which already affected results of accurate calculation of the target peak 2 (parathion-methyl). These results indicate that the developed method has a high accuracy and precision.

The developed method was then applied for the extraction and determination of four pesticide residues in actual carrot and yacon samples (Figures 6(d), 6(e) and Tables 4, 5). Parathion-methyl was not found in the carrot and yacon samples, indicating that this pesticide may not be widely applied in crop cultivation. Chlorpyrifos in yacon was quantitatively detected at a level of 1.26 ng·g^−1^ due to its wide application in planting, and phoxim in carrot was quantitatively detected at a level of 0.83 ng·g^−1^. Moreover, tolclofos-methyl was also, respectively, detected at two levels of 0.69 ng·g^−1^ in the carrot samples and 0.47 ng·g^−1^ in the yacon samples (Tables 4 and 5; Figures 6(d) and 6(e)). And all these detected values have not exceeded maximum residue limit (MRL) of the food of Japan and Korea.

Furthermore, compared with the published methods for the determination of organophosphorous pesticides in food, this developed method has easier popularization, less time consumption, lower LOD value, better linearity range, and easier peak identification. Thus, this method can facilitate the effective and efficient analysis of the phosphorothioate pesticides in food such as carrot and yacon samples.

## 4. Conclusions

An efficient method with high sensitivity, namely, molecularly imprinted matrix solid-phase dispersion coupled with GC, was developed for the rapid extraction and determination of four phosphorothioate organophosphorus pesticides in carrot and yacon. Under the optimized conditions, a high extraction efficiency was obtained for the four pesticides with low LODs (0.012~0.026 ng·g^−1^). Meanwhile, a good linearity of phosphorothioate pesticides was observed in a range of 0.05~17.0 ng·g^−1^, and the spiked recovery rates at two spiked levels were in a range of 85.4–105.6%. This method will provide a new tool for the rapid determination of multipesticide residues in the complicated food samples, which will facilitate the studies of food safety concerning carrot and yacon.

## Figures and Tables

**Table 1:** Adsorbance of imprinted polymer nanomicrospheres of three group (A, C-added magnesium chloride, B-no addition of magnesium chloride).

Adsorbant	Treated group	Concentration (mg·L^−1^)	0.04	0.2	0.4	1.0	2.0	4.0
		Pesticides	Actual adsorbance (*μ*g·g^−1^)					

Imprinted polymer microspheres	A	Tolclofos-methyl	7.2	36.1	77.3	140.8	162.4	173.2
		Chlorpyrifos	7.4	33.4	53.2	123.8	137.4	143.5
		Parathion-methyl	6.7	37.6	61.0	147.2	158.3	165.9
		Phoxim	7.7	38.9	48.1	118.6	147.2	161.3
	B	Tolclofos-methyl	4.6	28.3	43.1	107.3	122.9	129.4
		Chlorpyrifos	4.4	21.5	47.6	76.4	87.3	116.2
		Parathion-methyl	5.3	22.6	40.4	90.7	127.4	122.8
		Phoxim	4.2	25.2	44.7	82.7	96.4	111.7

Nonimprinted polymers microspheres	C	Tolclofos-methyl	6.6	18.0	32.3	47.2	50.7	51.3
		Chlorpyrifos	4.4	22.0	32.3	45.3	54.0	57.7
		Parathion-methyl	4.2	19.7	32.7	51.3	59.0	63.1
		Phoxim	5.1	23.3	29.0	39.2	49.3	68.2

Theoretical adsorbance (*μ*g·g^−1^)			8.0	40.0	80.0	200.0	400.0	800.0

MIPs: molecularly imprinted polymers; NIPs: molecularly nonimprinted polymers.

**Table 2:** Compared loading of four pesticides and iprobenfos by imprinted and nonimprinted polymers.

Pesticides	Sorbents	Initial solution (mg·L^−1^)	Final solution (mg·L^−1^)	*K*_*d*_	*K*	*K*′	*Q* (*μ*g·g^−1^)

Tolclofos-methyl	Imprinted	5	3.75	166.7	2.2	2.8	385
	Nonimprinted	5	4.52	53.1	0.8		

Parathion-methyl	Imprinted	5	3.82	154.5	2.0	1.9	270
	Nonimprinted	5	4.36	73.4	1.1		

Chlorpyrifos	Imprinted	5	4.43	64.3	0.8	1.6	120
	Nonimprinted	5	4.67	35.3	0.5		

Phoxim	Imprinted	5	3.94	134.5	1.7	2.0	262
	Nonimprinted	5	4.46	60.2	0.9		

Iprobenfos	Imprinted	5	4.33	77.4			30
	Nonimprinted	5	4.39	69.5			

Four pesticides (phoxim, tolclofos-methyl, chlorpyrifos, and parathion-methyl).

**Table 3:** Equation, correlation coefficients (*R*), and limit of detection (LOD) of the matrix solid-phase dispersion coupled with gas chromatography method.

Pesticide	Equation	*R*	LOD (ng·g^−1^)	Linear range (ng·g^−1^)

Phoxim	*y* = 235360*x* + 13640	0.9971	0.026	0.09~16.0
Tolclofos-methyl	*y* = 325471*x* + 37262	0.9993	0.017	0.07~13.0
Chlorpyrifos	*y* = 330378*x* + 23286	0.9996	0.021	0.09~17.0
Parathion-methyl	*y* = 24197*x* − 3517	0.9983	0.012	0.05~10.0

**Table 4:** Recovery rates of four pesticides in the spiked blank carrot samples when molecularly imprinted nanomicrospheres were used for extracting pesticides and results of actual samples (mean ± SD, *n* = 5).

Pesticides	Samples (ng·g^−1^)	Spiked level = 0.5 ng·g^−1^		Spiked level = 2.5 ng·g^−1^	
	Carrot	Detected content	Recovery (%)/RSD (%)	Detected content	Recovery (%)/RSD (%)

Phoxim	0.83	0.431 ± 0.026	86.2/(6.0)	2.29 ± 0.22	91.6/(9.6)
Tolclofos-methyl	0.69	0.445 ± 0.028	89.0/(6.3)	2.64 ± 0.23	105.6/(8.7)
Chlorpyrifos	N.D	0.452 ± 0.029	90.4/(6.4)	2.25 ± 0.19	90.0/(8.4)
Parathion-methyl	N.D	0.464 ± 0.034	92.8/(7.3)	2.43 ± 0.18	97.2/(7.4)

N.D: no detection; RSD: relative standard deviation.

**Table 5:** Recovery rates of four pesticides in the spiked blank yacon samples when molecularly imprinted nanomicrospheres were used for extracting pesticides and results of actual samples (mean ± SD, *n* = 5).

Pesticides	Samples (ng·g^−1^)	Spiked level = 0.5 ng·g^−1^		Spiked level = 2.5 ng·g^−1^	
	Yacon	Detected content	Recovery (%)/RSD (%)	Detected content	Recovery (%)/RSD (%)

Phoxim	N.D	0.447 ± 0.025	89.4/(5.6)	2.38 ± 0.22	95.2/(9.2)
Tolclofos-methyl	0.47	0.427 ± 0.021	85.4/(4.9)	2.31 ± 0.19	92.4/(8.2)
Chlorpyrifos	1.26	0.486 ± 0.024	97.2/(4.9)	2.28 ± 0.14	91.2/(6.1)
Parathion-methyl	N.D	0.506 ± 0.031	101.2/(6.1)	2.36 ± 0.16	94.4/(6.8)

N.D: no detection; RSD: relative standard deviation.
